# Computational prediction of protein-protein complexes

**DOI:** 10.1186/1756-0500-5-495

**Published:** 2012-09-09

**Authors:** Seema Mishra

**Affiliations:** 1 Department of Biochemistry, School of Life Sciences, University of Hyderabad, Hyderabad, AP, India

**Keywords:** Protein-protein complex prediction, Protein-protein interface, Unbound protein-protein docking, HHsearch, ZDOCK, ClusPro, MetaPPISP, Optimal docking area, Surface racer

## Abstract

**Background:**

Protein-protein interactions form the core of several biological processes. With protein-protein interfaces being considered as drug targets, studies on their interactions and molecular mechanisms are gaining ground. As the number of protein complexes in databases is scarce as compared to a spectrum of independent protein molecules, computational approaches are being considered for speedier model derivation and assessment of a plausible complex. In this study, a good approach towards *in silico* generation of protein-protein heterocomplex and identification of the most probable complex among thousands of complexes thus generated is documented. This approach becomes even more useful in the event of little or no binding site information between the interacting protein molecules.

**Findings:**

A plausible protein-protein hetero-complex was fished out from 10 docked complexes which are a representative set of complexes obtained after clustering of 2000 generated complexes using protein-protein docking softwares. The interfacial area for this complex was predicted by two “hotspot” prediction programs employing different algorithms. Further, this complex had the lowest energy and most buried surface area of all the complexes with the same interfacial residues.

**Conclusions:**

For the generation of a plausible protein heterocomplex, various software tools were employed. Prominent are the protein-protein docking methods, prediction of ‘hotspots’ which are the amino acid residues likely to be in an interface and measurement of buried surface area of the complexes. Consensus generated in their predictions lends credence to the use of the various softwares used.

## Findings

### Introduction

Protein-protein interactions (PPIs) form the hallmark of several biological processes. Recent years are witnessing the emergence of protein-protein complexes as prospective drug targets. Studies on protein-protein complexes in Protein Data Bank show distinction between complexes formed by identical (homocomplex) or non-identical (heterocomplex) protein molecules [1], between obligate and non-obligate (non-obligate are those heterocomplexes in which the interacting partners are not co-localized initially) complexes, and between transient and permanent complexes depending upon the complex’s lifetime; although many PPIs do not fall into distinct types [2]. Protein-protein contacts between these distinct types of complexes differ in terms of surface complementarities, steric, electrostatic, hydrophobic and hydrogen-bonding forces, accessible surface area, residue propensity and planarity [2,3].

Despite high-throughput experimental efforts in proteomics, the number of interacting protein complexes in databases remains low. *In silico* protein-protein interaction studies that were scarce earlier, primarily due to protein folding problem being impregnable to a practical solution, are gaining ground in recent times because of advances in the accuracy of prediction through computational tools. Here, an attempt has been made to develop an approach that can be utilized in computational protein-protein interaction studies between any two interacting protein heterocomplexes. As an example for the elucidation of ways and means towards *in silico* exploration, a hypothetical protein, HP986, found in *H. pylori* that binds to tumor necrosis factor receptor 1 (TNFR1) as observed by surface plasmon resonance [4] was studied.

A combination of several bioinformatics tools was implemented towards HP986-TNFR1 complex prediction. The softwares and web servers were carefully chosen based upon their wide use in literature as evidenced through PubMed search, their consistently high performance in Critical Assessment of Protein Structure Prediction (CASP) and Critical Assessment of PRediction of Interactions (CAPRI) community-wide comparative evaluations as well as some preliminary validation studies using known crystal structures of protein complexes. In this validation study, the programs Optimal Docking Area (ODA) and ZDOCK2.3 correctly identified the binding interface of some published experimental complex structures (Data not shown). Computational programs such as HHsearch/HHpred in SWISS-MODEL workspace for HP986 model generation, ZDOCK2.3 and ClusPro for unbound protein-protein docking, Optimal Docking Area and MetaPPISP for the prediction of interfacial residues and Surface Racer program for the calculation of buried surface area were used.

HHsearch/HHpred programs [5] implemented in SWISS-MODEL workspace [6] are sensitive techniques for remote homologue detection if high homology is not found between the target and template proteins. This is so because they are based on the pairwise comparison of profile hidden Markov models (HMMs). Profile HMMs contain information about the frequency of insertions and deletions at each column in addition to the amino acid frequencies in the columns of a multiple sequence alignment, thereby improving sensitivity significantly. Not surprisingly, in the recent-most CASP9 result, HHpred was ranked first among the automatic structure prediction servers in template-based modeling.

ZDOCK web server [7] has consistently performed well in several CAPRI rounds and is also implemented in the commercial Accelrys’ Discovery Studio software. Based upon Fast Fourier Transform correlation, this rigid-body protein-protein docking technique generates about 2000 complexes which can be clustered together using ClusPro [8] for ease of analyses. After complexes with favorable surface complementarities are retained, these are filtered to select those complexes with good electrostatic and desolvation free energies. ClusPro then generates cluster centers that are a representative set of complexes that form a cluster. The cluster centers are ranked according to cluster sizes.

MetaPPISP [9] and Molsoft’s Optimal Docking Area (ODA) [10] tools are used to predict the interfacial residues in a protein-protein complex. These two softwares are based on different algorithms and a consensus interface generated from these could be used to identify the possible docking site. Meta-PPISP is built up using cons-PPISP, Promate and PINUP individual servers, each using different attributes for prediction, hence representing a consensus. It uses an amino acid sequence as input and outputs a list of residues likely to be in an interface. ODA tool uses a 3dimensional (3D) structure as an input. It generates surface patches of different sizes in a protein and calculates the docking surface energy of these patches. This docking surface energy is based on atomic accessible surface area (ASA) of the component residues. In a recent paper published in Nucleic Acids Research, ODA was used to identify binding sites for spTranslin with itself as well as spTRAX which was supported by experimental evidence [11].

### Materials and methods

The protein structure predictions were done using web interfaces of the programs SWISSMODEL workspace and Phyre version 0.2 available in public domain. The protein 3D structures used in the study were downloaded from RCSB Protein Data Bank (PDB) website. All the softwares used here are listed in Table 1 alongwith their websites.

**Table 1:** Website addresses of the softwares and web servers employed in the studies

	
SWISS-MODEL workspace	http://www.swissmodel.expasy.org/workspace/
Phyre	http://www.sbg.bio.ic.ac.uk/phyre/
PDB	http://www.rcsb.org/pdb/home/home.do
Swiss PDB Viewer	http://spdbv.vital-it.ch/
PDBSum	http://www.ebi.ac.uk/pdbsum/
Jpred	http://www.compbio.dundee.ac.uk/~www-jpred/
Optimal Docking Area	http://www.molsoft.com/oda.html
MetaPPISP	http://pipe.scs.fsu.edu/meta-ppisp.html
Surface Racer	http://apps.phar.umich.edu/tsodikovlab/index_files/Page756.htm
ZDOCK	http://zdock.umassmed.edu/
ClusPro	http://nrc.bu.edu/cluster/

Molecular visualization and general analyses on the model were done using DeepView version 4.0 and Accelrys’ ViewerLite 4.2. For model validation (Ramachandran plot calculations), the PROCHECK tool available with PDBsum program was used. Secondary structure prediction was done with the program Jpred3.

Molsoft ICM Browser was used to visualize the ODA (Optimal Docking Area) identified for TNFR1 and HP986 model using the online ODA tool. The regions likely to be involved in an interface are denoted as red spheres whereas those not likely to be in an interfacial area are denoted as blue spheres. Protein-protein interaction site prediction was done using MetaPPISP. Calculations for minimization energy and interacting residues within 4.5 Å of those in another protein were done using DeepView version 4.0. Solvent accessible surface area (SASA) was calculated using the program Surface Racer 3.0. Buried surface area (BSA) was calculated according to the following formula: [SASA(Receptor) + SASA(ligand–SASA(receptor + ligand)]/2.

Protein-protein docking was performed with the web version of ZDOCK 2.3. In the crystal structure, the unliganded TNFR1 (PDB ID: 1NCF) exists as a dimer, and therefore only one molecule of TNFR1 (receptor) was taken for unbound protein-protein docking with HP986 model (ligand). The 2000 predictions returned by ZDOCK 2.3 were clustered using ClusPro to identify a representative set of complexes. 10 such complexes were returned with the highest ranking (first) complex representing the largest population size.

### Results and discussion

#### Protein structure prediction

Because the experimental 3-D structure of the HP986 protein is not available, the 3-D model was built using SWISS-MODEL in an automated mode. No significant hits with proteins in the database with a high homology level were identified in a simple BLASTp search. The template identified through the HHsearch method implemented in SWISS-MODEL workspace was 1XMXA (ExPDB code, ExPDB is a template library extracted from PDB, Protein Data Bank).

HHsearch/HHpred program implemented in SWISS-MODEL workspace works as follows: To detect distantly related template structures, a target sequence can be searched against a hidden Markov model (HMM) based template library. Each HMM of the library is based on a multiple sequence alignment of the template sequence built by PSI-BLAST search enriched with secondary structure assignment. In the latest Critical Assessment of Protein Structure Prediction 9 (CASP9) result, HHpred was ranked first in automatic structure prediction servers in template-based modeling, thereby enhancing confidence in the model’s reliability. This template was also identified consistently using Phyre structure prediction program [12].

**Figure 1:**
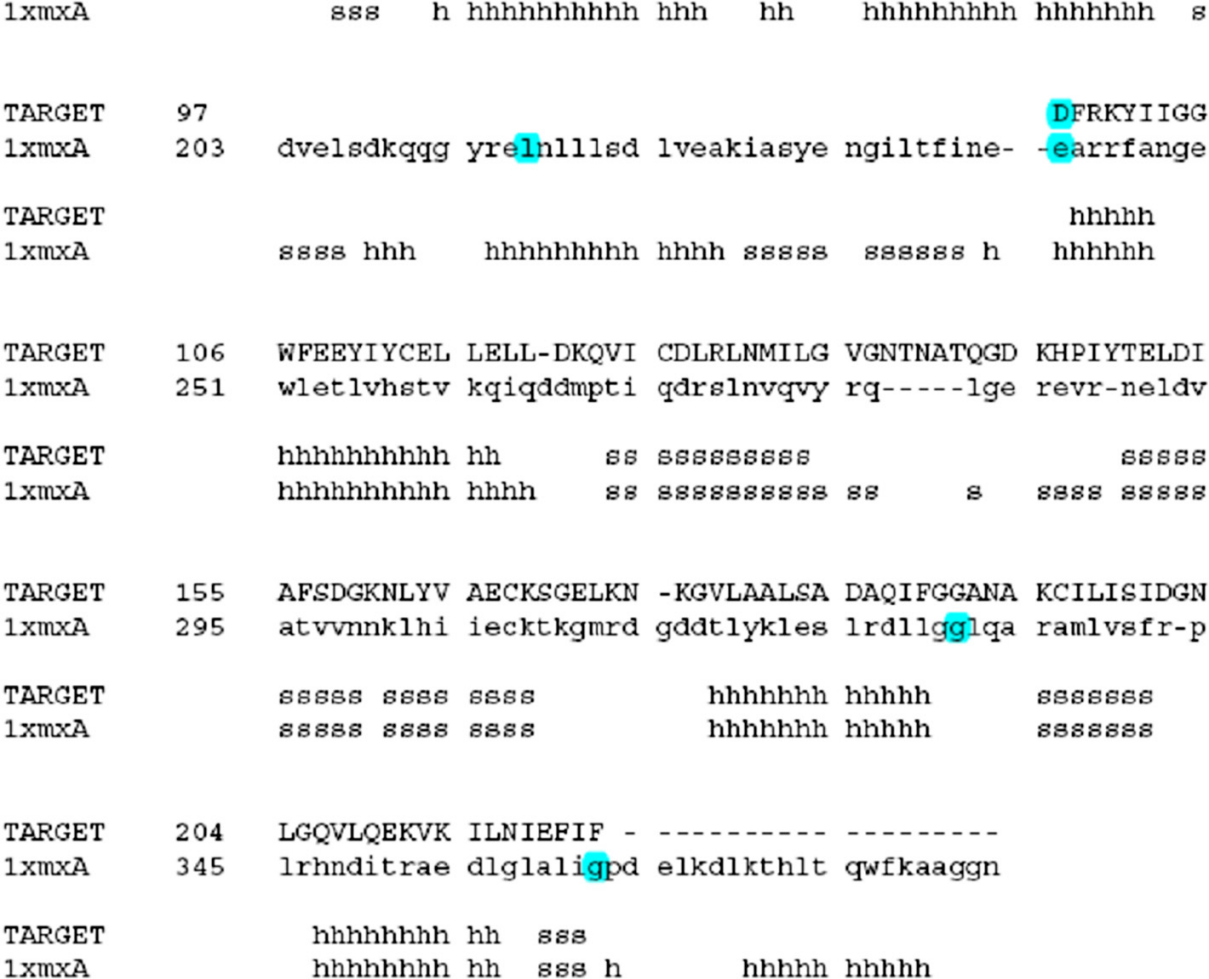
**An alignment of the target (HP986) and template (VC1899 protein, PDB ID 1XMX) generated by SWISS-MODEL.** Helices and sheets are represented by alphabets ‘h’ and ‘s’, respectively.

**Figure 2:**
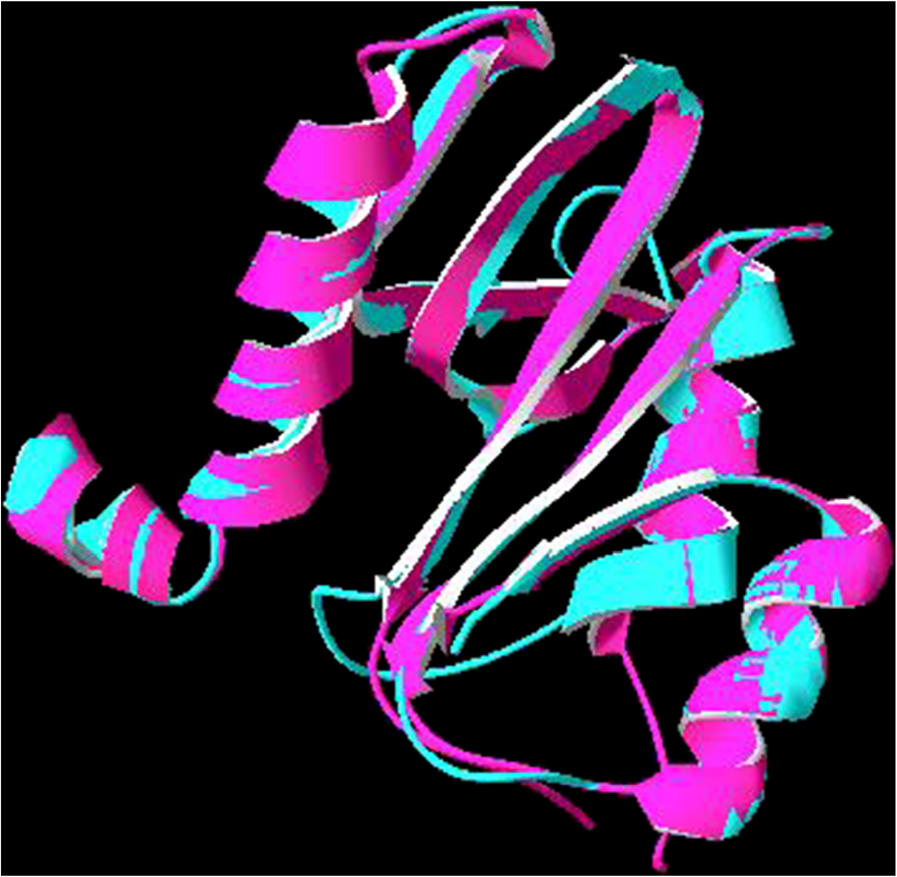
Superimposed structures of modelled HP986 protein (Blue) and VC1899 protein (Magenta).

1XMX is a hypothetical protein named VC1899 from Vibrio cholerae. The sequence identity between HP986 and VC1899 is 22%. The PROCHECK [13] score after re-building of two loops in the modelled region followed by subsequent energy minimization was 90.9% in most favored and 1.8% in disallowed regions, whereas before it was 88.2% in most favored regions and 3.6% in disallowed regions. Gaps in the alignment file generated (Figure 1) were negligible. The target and template structures after superimposition are shown in Figure 2. The secondary structure motifs for HP986 were verified from an independent secondary structure prediction tool, Jpred [14]. The Jpred predictions in the modeled region were consistent with the alpha-alpha-beta-beta-beta-alpha-beta-alpha-beta secondary structure prediction returned by SWISS-MODEL workspace. 

#### Docking simulations

The unbound protein-protein docking was carried out using ZDOCK2.3 with default parameters. 2000 predictions were generated using TNFR1 (PDB ID 1NCF) as receptor and HP986 model as ligand. The 2000 complexes generated from ZDOCK were submitted to ClusPro in order to cluster them. 10 cluster centers were returned by ClusPro, with the first ranked cluster center containing the highest number of complexes.

**Figure 3:**
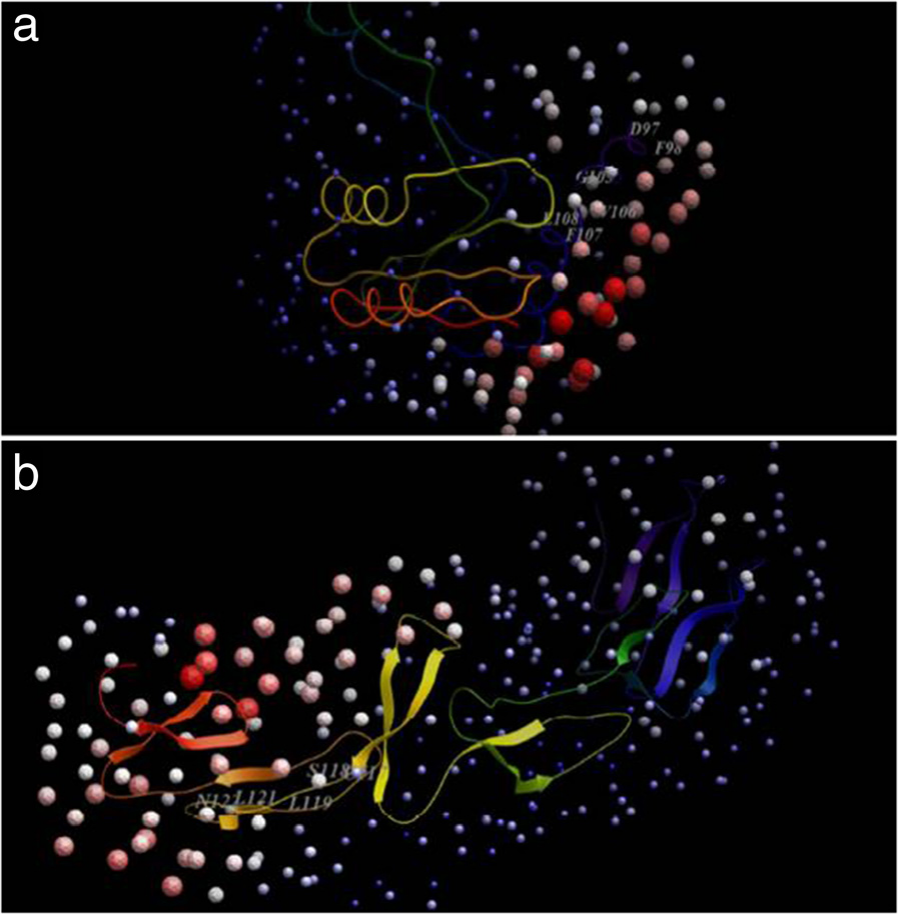
**Likely hotspots in HP986 (a) and TNFR1 (b) as identified by Optimal Docking Area tool.** The regions denoted by light red spheres likely to be involved in an interface are labeled with first few residues each of which is identified by MetaPPISP also.

The next step was to identify the interfacial area where these two proteins are likely to bind. There is no mutation data in the literature for identifying the binding interface. Hence, to generate data for likely interaction site, a list of interface residues common to the results returned by MetaPPISP and ODA tool for both HP986 and TNFR1 proteins was made (Table 2, Figures 3a and b). This list was used to analyse the 10 docked complexes for the presence of such residues in the interface. Most of the interfacial residues were present in the first ranked complex forming the largest size cluster, as well as the complexes ranked 5 and 10. Other complexes contained HP986 binding at a different location on TNFR1, and residues in this location were not predicted as interfacial residues by Meta-PPISP and ODA. Hence, these complexes were not taken into account for analyses further.

**Table 2:** A list of putative interacting residues in the protein-protein interface for TNFR1 and HP986 proteins using a combination of Meta-PPISP and ODA tools

**TNFR1**	**HP986**
C104(117), S105(118), L106(119), L108(121),N109 (122), T111(124) H113(126), L114(127), C116(129), N121(134) to E136(149)	D1(97), F2(98), R3(99), K4(100), Y5(101), I6(102), I7(103), G9(105) to F11(107), E13(109), Y14(110), Y16(112) to E18(114), L20(116), R32(128) to I36(132), K72(168), L76(172), I104(200), D105(201), I124(220)

Residue numbers are numbered according to the model returned by ZDock and ClusPro. (Numbers in parentheses are the numbers in the actual sequence in NCBI database).

There were no steric clashes, since the complexes are subjected to CHARMm minimization by the ClusPro program itself. However, the complexes 1, 5 and 10 were further subjected to a short minimization using DeepView with Gromos96 force field *in vacuo* and the minimization energy score was determined for comparison. Buried surface area (BSA) of the complexes was calculated using Surface Racer program [15] to identify the complex having the largest contact area between the two proteins.

Table 3 shows the minimization energy values, buried surface area and interacting residues for the three complexes 1, 5 and 10. Interacting residues here have been calculated using DeepView to ascertain those residues in HP986 that are within 4.5 Å of same TNFR1 residues in all the three complexes. As seen from the table, while complex 5 has the lowest energy and most buried surface area as calculated using Chothia (1976) van der Waals radii set, complex 1 has more buried surface area as calculated using Richards (1977) van der Waals radii set implemented in Surface Racer. Only those interacting residues are listed which are also present in the list generated by Meta-PPISP and ODA tool. It is seen that complex 5 has the most number of interacting residues.

**Table 3:** **Minimization energy (in kJ/mol) and buried surface area (in Å**^**2**^**) values and interacting residues within 4.5 Å of residues in another protein in three complexes returned by ClusPro**

**Complex**	**Minimization Energy (kJ/mol)**	Buried Surface Area* (Å^**2**^**)**	Interacting Residues**	Interacting Residues**
			**TNFR1**	**HP986**
Complex 1	−22354.95	1194.3 (4867.7)	C104, S105, L106, L108, N109, T111, H113, L114, C116	R3, I7, L76
Complex 5	−22624.33	1218.7 (4792.9)	Same as above	D1, R3, K4, Y5, I7, G9, W10, E13, R32, L33, N34, M35, I36, L76
Complex 10	−22538.17	1193.7 (4826.6)	Same as above	L76

*The numbers represent BSA calculated using Chothia (1976) van der Waals radii set, while those in parentheses represent BSA as calculated using Richards (1977) van der Waals radii set using Surface Racer program.

**The interacting residues here have been calculated using DeepView to identify those residues in HP986 that are within 4.5 Å of same TNFR1 residues in the complexes. Only those residues which are also present in the list generated by Meta-PPISP and ODA tool are listed.

**Figure 4:**
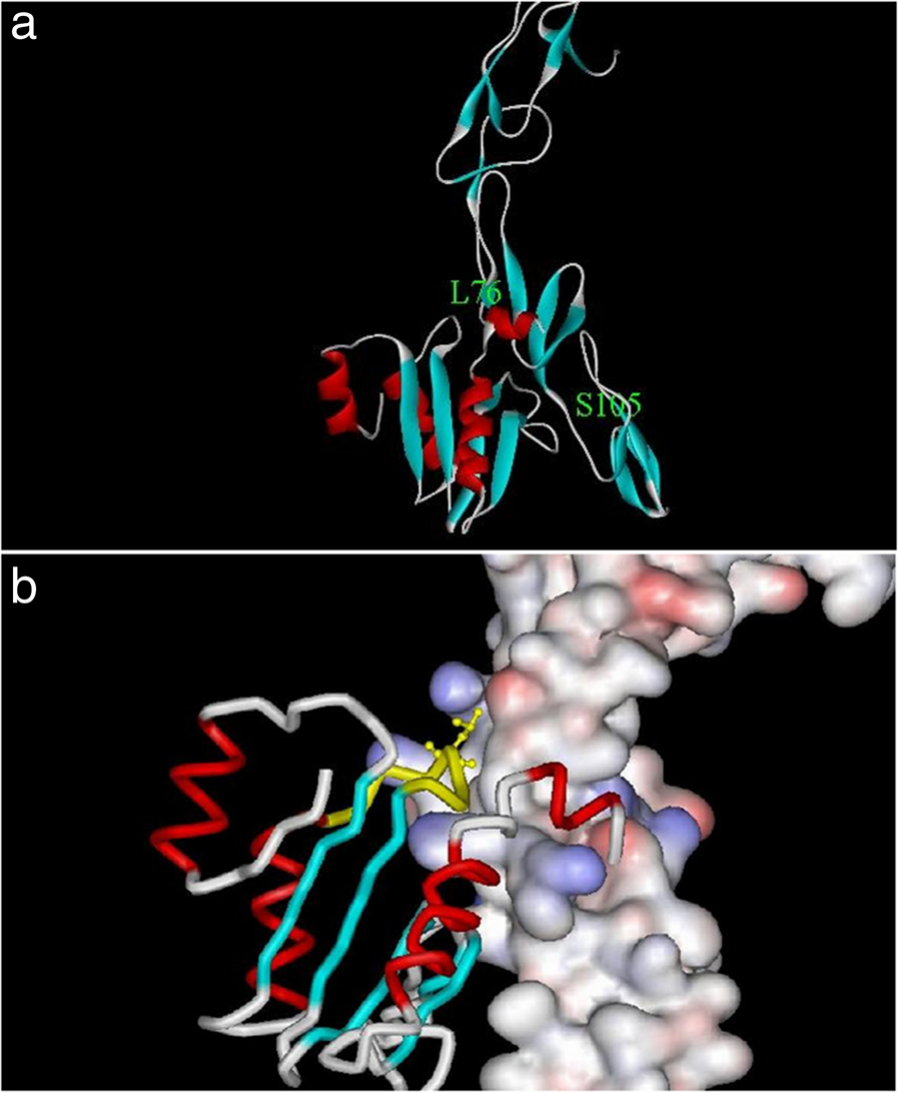
**a: A ribbon representation of complex 5 returned by ClusPro using ZDOCK-generated complexes as input.** The location of a residue each on TNFR1(S105 in the elongated, all-beta structure) and HP986 (L76 in the alpha + beta structure) is shown as an example to demarcate the likely binding site. **b:** TNFR1 is rendered as molecular surface colored with electrostatic potential and HP986 is rendered in tube representation. The loop region containing L76 (172) residue is colored in yellow. L76 residue is shown as a ball-and-stick model.

It is evident from the multiple results generated that among all the three candidates, complex 5 has emerged as the most plausible candidate. Figure 4a shows a ribbon representation of complex 5 returned by ClusPro. In Figure 4b, TNFR1 is shown rendered as molecular surface colored with electrostatic potential and HP986 is rendered as tube representation. This complex can be used for further studies such as intermolecular interaction analyses providing newer hypotheses.

### Conclusions

This paper delves on the approach taken towards the prediction of the most plausible protein-protein heterocomplex from thousands of complexes generated and in the event of little or no information available for the interface of the two binding partners. It is interesting to note that all the different prediction tools used here, with either the sequence or the structure as inputs, were consensual in the results generated. This lends greater confidence in the approach used. Structurally, HP986 protein domain seems to belong to alpha + beta protein fold family, whereas its interacting partner, TNFR1, is an elongated all-beta structure. Simulations to model the conformational changes of interacting proteins may include molecular dynamics studies on protein mutants that provide a valuable insight into the investigation of conformational behaviour and dynamics of a particular protein [16]. The question of the accuracy of complex prediction remains dependent on the experimental verification. There are reports on the experimental verification using these tools, a recent one is presented by Eliahoo *et al.* (2010) [11]. The approach taken here can be utilized towards the *in-silico* characterization of any protein-protein hetero-complex which can help generate hypotheses for experimental work later on.

## Abbreviations

HP986: Hypothetical protein found in *Helicobacter pylori*; TNFR1: Tumor necrosis factor receptor 1; spTranslin: *Schizosaccharomyces pombe* protein translin; spTRAX: Translin paralog associated with translin; VC1899: Hypothetical protein from *Vibrio cholerae*.

## Competing interests

The author(s) declare that they have no competing interests.
